# *Chrysosporium*-Related Fungi and Reptiles: A Fatal Attraction

**DOI:** 10.1371/journal.ppat.1004367

**Published:** 2014-10-16

**Authors:** F. Javier Cabañes, Deanna A. Sutton, Josep Guarro

**Affiliations:** 1 Veterinary Mycology Group, Department of Animal Health and Anatomy, Veterinary School, Universitat Autònoma de Barcelona, Bellaterra, Catalonia, Spain; 2 Department of Pathology, University of Texas Health Science Center, San Antonio, Texas, United States of America; 3 Mycology Unit, School of Medicine and Health Sciences, Institut d'Investigació Sanitària Pere Virgili, Universitat Rovira i Virgili, Reus, Catalonia, Spain; Duke University Medical Center, United States of America

## The Genus *Chrysosporium*: Its Clinical Importance

The anamorphic (asexual) genus *Chrysosporium* Corda includes mostly keratinophilic species that live on the remains of hair and feathers in soil. These fungi are rarely reported as animal pathogens, apart from in reptiles, and only a few species have been involved in mycoses. In a comprehensive review of opportunistic mycoses published by Smith [1], only one case of a *Chrysosporium*-incited cutaneous abscess in a snake was cited with no additional details provided. A few other cases have been published in a small range of animal species including *C. pannicola* (formerly *C. evolceanui*) from affected skin of a dog [2], a case of keratomycosis in a horse [3], a probable case of mycosis caused by *C. tropicum* in two breeds of chickens [4], and a disseminated infection in a dog [5] by a pair of *Chrysosporium* isolates not identified to the species level. Some species also occasionally infect humans with reports of *C. keratinophilum* and *C. pannicola* in skin and nail infections and some deep infections by *C. zonatum* [6]. However, in recent years there has been a noticeable increase in mycoses caused by some *Chrysosporium*-related fungi in reptiles. These fungal pathogens are, however, unrelated to the chytrids (Phylum: Chytridiomycota), such as *Batrachochytrium dendrobatidis*, which is causing the current catastrophic die-off of amphibians, i.e., chytridiomycosis in frogs [7].

## *Chrysosporium*-Related Fungi: Incidence, Pathogenicity, and Potential Causes of Disease in Reptiles

**Figure 1:**
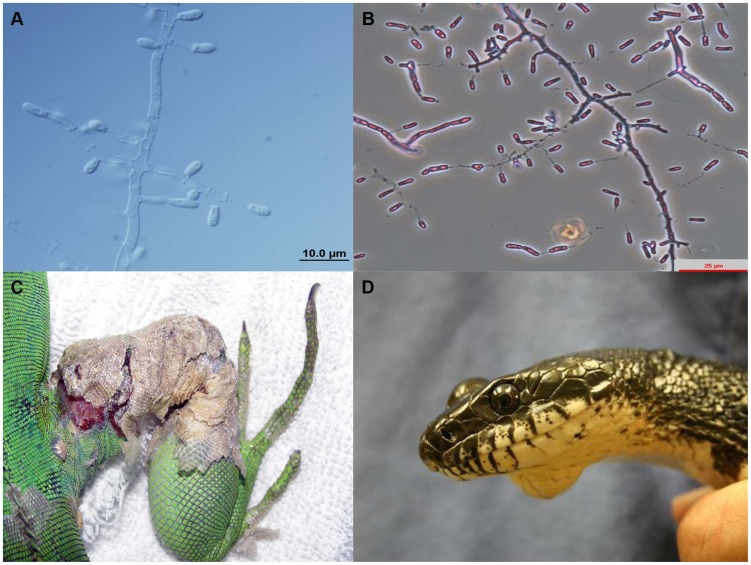
Microscopic morphology of *Nannizziopsis guarroi* (A) and *Ophidiomyces ophiodiicola* (B). Green iguana showing ulcerative dermatitis in the right leg caused by *N. guarroi* (C). Black rat snake showing a mycotic granuloma caused by *O. ophiodiicola* (D). Figure 1D was published previously by Rajeev et al. [22].

Organisms identified as the *Chrysosporium* anamorph of *Nannizziopsis vriesii* (unsuitably abbreviated as CANV in some reports) and similar undescribed species [8]–[18], *C. guarroi* [19], [20] (now a synonym of *Nannizziopsis guarroi*; Figure 1A [21]) and *C. ophiodiicola* [22] (now a synonym of *Ophidiomyces ophiodiicola*; Figure 1B [23]) have been isolated with some frequency from reptiles in recent years. These fungi are the cause of superficial and deep mycoses that affect pets as well as captive and wild animals. Several reports indicate that these organisms are an emerging cause of fungal disease in bearded dragons and iguanas [8], [10], [11], [19], [24]. As these species of reptiles continue to gain popularity as pets, the disease is being found worldwide with cases reported thus far in Asia, Australia, Europe, and North America. The source(s) of the etiologic agents of this contagious mycosis, however, are yet unknown. One survey of the skin of healthy squamate (covered with scales) reptiles from zoological and veterinary institutions revealed that *Chrysosporium*-related fungi are present in very low numbers in the cutaneous mycobiota of reptiles [25].

Bearded dragons kept in captivity suffer a sometimes fatal dermatological condition known in the pet trade as “yellow fungus disease,” and it has been suggested recently that *N. guarroi* and some other related species are the etiological agents [10], [21], [23]. The circumstances under which mycotic diseases in these species occur are not yet known, although inadequate diet and husbandry, environmental stresses, trauma, and existing dermatitis are all likely contributors [26]. Furthermore, some *Chrysosporium*-related fungi, especially *N. dermatitidis*, can act as primary pathogens in veiled chameleons [23], [27]. Infection by these species in bearded dragons begins as a cutaneous disease often characterized by vesicular lesions and bullae. Necrosis, sloughing, and ulceration then follow, progressing to involve muscle and bone. The infection can disseminate with a fatal outcome [10], [12], [23], [28]. In captive and pet green iguanas, cases of superficial dermatomycoses and other more severe infections that progress to involve muscle and bone have been recently reported [11], [19], [20]. Most of these cases were probably caused by *N. guarroi* (Figure 1C) [21], [23]. Table 1 summarizes other *Chrysosporium*-related fungi involved in cutaneous and/or systemic mycoses in a variety of other captive and wildlife reptile species (Figure 1D). Infections caused by *Nannizziopsis*, *Paranannizziopsis*, and *Ophidiomyces* species are contagious among reptiles [23].

**Table 1:** Proposed species causing infection in reptiles [21], [23].

*Chrysosporium*-related fungi	Reptile species
***Nannizziopsis*** Currah (1985)	
*N. arthrosporioides* Stchigel et al. (2013)	Water dragon (*Physignathus* sp.)
*N. barbata* Sigler et al. (2013)	Coastal bearded dragon (*Pogona barbata*)
*N. chlamydospora* Stchigel et al. (2013)	Inland bearded dragon (*Pogona vitticeps*)
*N. crocodili* Sigler et al. (2013)	Saltwater crocodile (*Crocodylus porosus*)
*N. dermatitidis* Sigler et al. (2013)	Chameleons, geckos
*N. draconii* Cabañes et al. (2013)	Inland bearded dragon
*N. guarroi* Cabañes et al. (2013)	Green iguana (*Iguana iguana*), inland bearded dragon, lizard (*Agama agama*), snake
*N. pluriseptata* Stchigel et al. (2013)	Skink lizard (*Eumeces inexpectatus*)
*N. vriesii* Currah (1985)	Lizard (*Ameiva* sp.)
***Paranannizziopsis*** Sigler et al. (2013)	
*P. australiensis* Sigler et al. (2013)	Northern tuatara (*Sphenodon punctatus punctatus*), coastal bearded dragon, aquatic file snake (*Acrochordus* sp.)
*P. californiensis* Sigler et al. (2013)	Tentacled snake (*Erpeton tentaculatum*)
*P. crustacea* Sigler et al. (2013)	Tentacled snake
*P. longispora* Sigler et al. (2013)	Tentacled snake
***Ophidiomyces*** Sigler et al. (2013)	
*O. ophiodiicola* Sigler et al. (2013)	Snakes

A recent essay [29] hypothesized that fungal proliferation after the devastation of the Cretaceous-Tertiary (K-T) event preferentially selected for the fungal-resistant endothermic mammals and hindered the reemergence of a second reptilian age. The darkened skies and cooler temperatures that accompanied the K-T cataclysm would have shielded the sun and reduced the ability of ectothermic creatures such as reptiles to induce fevers by insolation, a necessary activity for protection against fungal diseases. Historically, mycotic infections in reptiles have likely remained underdiagnosed. Most recognize fungal infections as secondary infections resulting primarily from poor husbandry and underlying chronic comorbidities. Along with good food and proper husbandry, adequate light and heat are also essential to reptile health [30].

## *Chrysosporium*-Related Fungi: Taxonomy

The genus *Chrysosporium* is polyphyletic, having affiliation with at least two orders of the Ascomycota; however, rDNA sequencing studies which included a representative number of reference *Chrysosporium* and related species indicate that it should be restricted to anamorphs (asexual states) in the order Onygenales [31]. Relevant fungal pathogens that produce important mycoses such as blastomycosis, coccidioidomycosis, dermatophytosis, histoplasmosis, and paracoccidioidomycosis are also grouped in this order. These species usually produce white to yellowish colonies and poorly differentiated fertile hyphae with terminal, lateral, and usually one-celled conidia. These conidia are broader than the diameter of the supporting hyphae and are mainly clavate or pyriform with a truncated base, sessile or borne on short protrusions or side branches of the vegetative hyphae [6]. Species of *Chrysosporium* are not easy to identify since their conidia are similar to those of other anamorphic genera such as *Blastomyces*, *Emmonsia*, *Geomyces*, *Malbranchea*, and *Myceliophthora*, and also to some species in the dermatophyte genus *Trichophyton* that produce only microconidia. About 65 *Chrysosporium* species are currently accepted and their sexual morphs (teleomorphs) are found in a variety of genera such as *Aphanoascus*, *Arthroderma*, or *Nannizziopsis*, among others [32].

*Nannizziopsis vriesii* (Apinis) Currah (Ascomycota, Onygenales, Onygenaceae) has white ascomata, asperulate peridial hyphae constricted at septa, hyaline and globose ascospores, and a *Chrysosporium* asexual morph. The ex type strain of this species was isolated from the skin and lungs of a lizard [33], [34]. Most fungal isolates from reptiles have been considered to belong to the *Chrysoporium* anamorph of *N. vriesii* because of morphological similarities of the anamorph with those of this ascomycete. However, no sexual structures of *N. vriesii* have been obtained in these case reports and some phenotypic and molecular differences among isolates from reptiles have been detected. This corroborates what was suggested several years ago from preliminary molecular phylogenetic analysis that the *Chrysoporium* anamorph of *N. vriesii* actually represented a species complex, rather than a single species, containing members that could be allied to specific hosts [26].

Recently, Stchigel et al. [21] and Sigler et al. [23] published the latest taxonomic revisions regarding *Chrysosporium*-related fungi, also noting relationships between specific fungal species and different reptile hosts. These new revisions also comply with the recent changes in the International Code of Nomenclature for algae, fungi, and plants (one fungus, one name) [35]. In these papers [21], [23], one new family (Nannizziopsiaceae), two new genera (*Ophidiomyces* and *Paranannizziopsis*), and 15 new species were proposed. Table 1 highlights these new *Chrysosporium*-related fungi and the reptile hosts they are known to infect.

Given the difficulty in identifying *Chrysosporium* species morphologically, and prior to our knowledge of closely related genera, several older reports of infection in reptiles by species such as *C. keratinophilum* and *C. tropicum* [17] may have actually been caused by the *Chrysosporium* anamorph of *N. vriesii*. Another example is the *C. queenslandicum* isolate from a case report of a mycosis in a snake [36], which upon reexamination of the fungus was indeed found to be the *Chrysosporium* anamorph of *N. vriesii* [28].

## Treatment of *Chrysosporium*-Related Infections in Reptiles

Treatment of fungal infections in reptiles includes the administration of effective antifungal agents for a minimum of 2 to 4 weeks, together with the correction of inappropriate environmental conditions. As most cases of mycotic diseases in reptiles are diagnosed at necropsy, there are relatively few reports that discuss effective dosages and dosage intervals of antifungal agents. The systemic drugs of choice for use in reptiles diagnosed with infection caused by filamentous fungi include ketoconazole and itraconazole [26]. Voriconazole seems to be also a safe and effective antimycotic drug to eliminate these infections in bearded dragons [24]. However, treatment using antifungals has shown mixed results [37]. As mentioned earlier, adequate light and heat are also essential to reptile health and largely influence clinical recovery, given that the reptile's immune response, metabolism of drugs, and use of fluid therapy are heat dependent [30].

## Human Infections Caused by *Chrysosporium*-Related Fungi Affecting Reptiles

As mentioned above, it has been demonstrated that various *Chrysosporium*-related fungi appear to be host specific. This is evidenced by the fact that three species of *Nannizziopsis* which have occasionally infected humans or have been found in clinical samples in the United States have never been implicated in reptile disease [23]. These species include *N. hominis*, recovered from groin lesions, inguinal nodes, and leg abscesses of an HIV-positive patient and from an inguinal node of an immunocompetent patient with disseminated adenopathy, *N. infrequens*, isolated from a bronchial wash specimen of an HIV-positive patient, and *N. obscura* in a case of osteomyelitis [21], [23]. This somewhat mitigates zoonotic concerns associated with handling popular pet reptiles such as green iguanas or bearded dragons, in which *N. guarroi* is the common etiologic agent [23]. However, the identity of a *Chrysosporium*-related isolate that produced a lung infiltration and a brain abscess in a Nigerian HIV-positive patient in Germany [38] is still unclear. It was originally identified as *Chrysosporium* anamorph of *N. vriesii*, but phenotypic and molecular characteristics of the isolate were not provided by the authors [38]. This isolate has been recently identified as an atypical strain of *N. vriesii* by Stchigel et al. [21] and as a strain close to *N. obscura* by Sigler et al. [23]. Most of these few human cases occurred as opportunistic infections in immunocompromised patients. In regards to infection, handling pets is no more risky for an immunosuppressed person than is contact with other people or the environment [39]. However, in these cases, a special precaution should be taken because of the fact that exotic or wild animals may harbor unusual pathogens.
